# Improved Side-Channel Attack on CTR DRBG Using a Clustering Algorithm

**DOI:** 10.3390/s25134170

**Published:** 2025-07-04

**Authors:** Jaeseung Han, Dong-Guk Han

**Affiliations:** Department of Financial Information Security, Kookmin University, Seoul 02707, Republic of Korea; jae1115@kookmin.ac.kr

**Keywords:** AES, block cipher, counter mode, DRBG, side-channel attack

## Abstract

Deterministic random bit generators (DRBG) play a crucial role in device security because they generate secret information cryptographic systems, e.g., secret keys and parameters. Thus, attacks on DRBGs can result in the exposure of important secret values, which can threaten the entire cryptographic system of the target Internet of Things (IoT) equipment and smart devices. In 2020, Meyer proposed a side-channel attack (SCA) method that recovers the output random bits by analyzing the power consumption traces of the NIST standard AES CTR DRBG. In addition, most algorithmic countermeasures against SCAs also utilize random numbers; thus, such vulnerabilities are more critical than other SCAs on cryptographic modules. Meyer’s attack recovers the secret random number in four stages of the attack using only the power traces, which the CTR DRBG processes in 256 blocks. We present an approach that employs a clustering algorithm to enhance Meyer’s attack. The proposed attack increases the attack success rate and recovers more information using a clustering attack in the first step. In addition, it improves the attack accuracy in the third and fourth steps using the information obtained from the clustering process. These results lead to the possibility of attacks at higher noise levels and increase the diversity of target devices for attacking the CTR DRBG. Experiments were conducted on an Atmel XMEGA128D4 processor to evaluate the effectiveness of the proposed attack method. We also introduced artificial noise into the power traces to compare the proposed attack’s performance at different noise levels. Our results demonstrate that the first step of the proposed attack achieves a higher success rate than Meyer’s attack at all noise levels. For example, at high noise levels, the difference in the success rates is up to 50%. In steps 3 and 4, an average performance improvement of 18.5% greater than Meyer’s proposed method is obtained. The proposed attack effectively extends the target to more noisy environments than previous attacks, thereby increasing the threat of SCA on CTR DRBGs.

## 1. Introduction

Side-Channel Attacks (SCAs) target cryptographic algorithms by analyzing the channel information from the target devices. SCAs have been used to attack embedded devices using various channel information, e.g., power consumption [1], timing [2], electromagnetic emission [3], photonic emission [4], and fault injection [5], in various platforms, including software [6,7,8] and hardware implementations [8,9,10]. In 1996, Kocher presented a timing attack for the first SCA method and recovered the secret key of public key cryptography by applying the timing attack [11]. Then, differential power analysis (DPA) [1], which is an SCA technique that uses the difference in the power consumption, correlation power analysis (CPA) [12] using Pearson’s correlation coefficients (PCC) between the actual and hypothetical power consumption, and mutual information analysis (MIA) [13] using the mutual information between the actual and hypothetical power consumption were proposed. Recently, differential deep learning analysis (DDLA) using the difference in deep learning results was proposed [14]. In addition, template attacks [15] that construct templates using a profile device, deep learning-based profiled SCA [16], and SCA using clustering algorithms were presented [17,18,19].

SCA methods have been applied to block ciphers and their modes of operation. For example, CPA and SPA have been applied to the modes of operation of AES, ECB, CBC, and CFB [20,21,22,23,24,25,26,27,28]. Note that most of these attacks require specific information, e.g., plaintexts, ciphertexts, and initial vectors; however, in 2007, Jaffe proposed a power analysis technique that can recover the master key of the AES counter (CTR) mode using 65,536 power traces without knowledge of the initial counter, input, or output of the CTR mode [26]. Then, in 2020, Meyer proposed an improved power analysis that required only 256 power traces to attack the AES CTR mode [27]. This attack causes a new attack in another application of AES. She demonstrated that the attack could be applied to the AES CTR DRBG because the required number of traces (blocks) of this attack, i.e., 256, is less than the CTR DRBG’s number of the least key life cycle traces (blocks), i.e., 4096. In 2023, Tienteu et al. presented a template attack against the AES CTR mode [28].

In Internet of Things (IoT) devices, CTR DRBG is primarily used to generate cryptographic secret information, e.g., secret keys, and protocols related to CTR DRBG, and optimal implementations in IoT environments have been studied previously [29,30,31,32]. The physical security evaluation of CTR DRBG is important relative to the security of cryptographic systems in IoT environments and is crucial from the SCA perspective. In masking and hiding [8], which are representative SCA countermeasures, secret randomness is employed, and the leakage of this randomness directly leads to the nullification of the countermeasures. However, if such SCA countermeasures are applied to DRBG, a circular problem will occur in which randomness is used to ensure the security of DRBG that generates randomness.Therefore, the physical security level should be evaluated and countermeasures should be discussed through SCA research on CTR DRBG in IoT environments by physical noise level.

From the perspective of the CTR DRBG attacker, Tienteu’s attack requires a stronger capability than Meyer’s. In addition, Tienteu’s template attack requires a profiling device that can control the DRBG’s internal state information; however, Meyer’s attack does not require a profiling device and, similar to Jaffe’s attack, only uses the power consumption to recover the internal secret value. In CTR DRBG attacks, unlike in general SCA scenarios, the number of traces an attacker can obtain is strictly limited by the DRBG’s internal state update cycle. Thus, whether an attack on a target is possible is determined by the performance of the attack method, and the attack success rate under the same conditions (e.g., the noise level and number of traces) becomes much more important than in other SCA scenarios.

This paper presents an improved SCA method for AES CTR DRBG based on Meyer’s approach. The proposed method employs a clustering algorithm and achieves a higher success rate than Meyer’s approach for all noise levels.

### 1.1. Contribution

**Figure 1:**
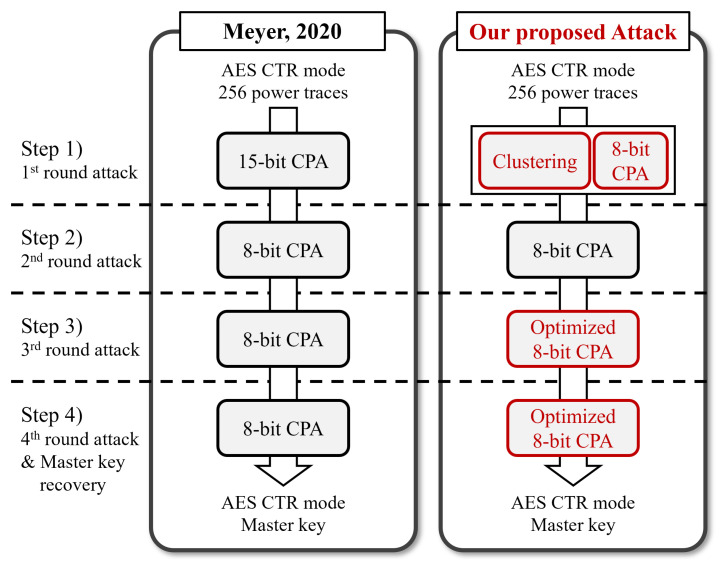
Summary of Meyer’s attack [27] and the proposed attack process.

Figure 1 summarizes the processes of Meyer’s approach and the proposed attack. CPAs can be replaced with other non-profiled SCA methods, e.g., DPA, MIA, and DDLA, in these attack processes. We describe those based on CPA in this study. This paper presents an enhanced power analysis attack on the AES CTR DRBG that outperforms previous methods. Building on Meyer’s approach, the proposed attack method uses only 256 power traces and comprises four main steps. In step 1, we employ a clustering algorithm and an 8-bit CPA to recover 1 byte of each CTR mode IV value and the AES key. Our method recovers more information than Meyer’s attack, totaling 16 bits. The second step is the same as Meyer’s. In the third and fourth steps, we perform a more accurate CPA using the additional information obtained in the first step.

We apply the proposed and Mayer’s attacks on the Atmel XMEGA128D4 processor environment and compare their success rates. In addition, we compare the attacks at various noise levels by introducing artificial noise. The results demonstrate that the first step of the proposed attack exhibits a higher success rate than Mayer’s attack at all noise levels, particularly at high noise levels, with a success rate difference of up to 50%. Furthermore, the computational complexity of the first phase of our analysis is 128 times lower. In steps 3 and 4 of the attacks, the CPA accuracy of the proposed attack is approximately 1.18 times higher than that of Meyer’s attack.

### 1.2. Organization

The remainder of the paper is organized as follows. Section 2 defines the notations used in this paper and provides relevant background information, including the AES CTR mode, the CPA process, and the clustering algorithm. Section 3 summarizes Meyer’s attack. Section 4 proposes a novel attack on the AES CTR mode. Section 5 demonstrates the first step of the proposed attack on the Atmel XMEGA128D4 processor, and Section 6 compares the performance of the proposed attack with Meyer’s attack. Finally, the paper is concluded in Section 7, including a brief discussion of countermeasures.

## 2. Preliminaries

### 2.1. AES CTR Mode

AES is a 128-bit block cipher that uses a substitution-permutation network structure [33]. It offers different versions, e.g., AES-128, AES-192, and AES-256, based on the master key length. The number of rounds for AES-128, AES-192, and AES-256 is 10, 12, and 14, respectively. The AES round comprises four functions, i.e., AddRoundKeys, SubBytes, ShiftRows, and MixColumns functions.

**Figure 2:**
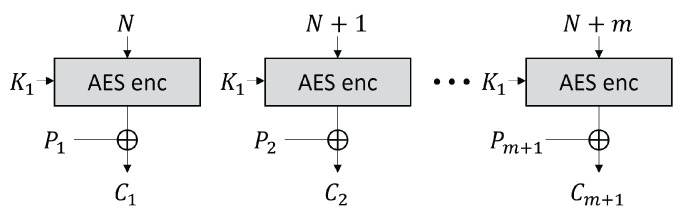
AES CTR mode of operation.

**Figure 3:**
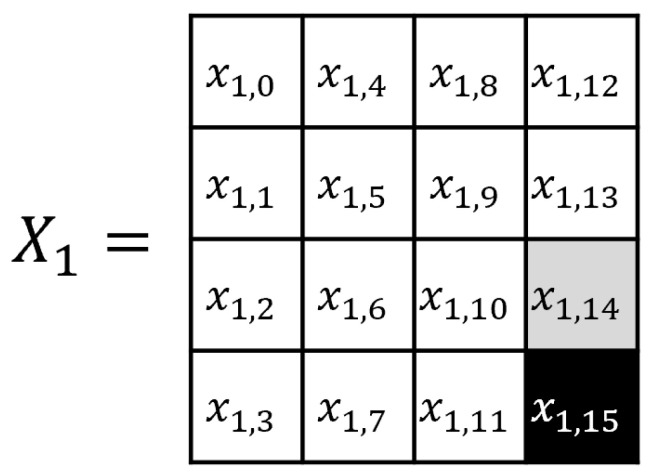
AES input state in the CTR mode.

The CTR mode is a mode of operation for the encryption and decryption of multiple blocks through a block cipher [34]. The CTR mode uses an initial nonce, and each (m+1)-th block is encrypted using the exclusive or (XOR) operation with the block cipher encryption result of (N+m). Figure 2 shows the AES CTR mode of operation when the input block length is m+1, and Figure 3 shows the AES encryption input in the CTR mode, where the black cell represents a byte that changes at each 256 AES input block in the CTR mode, and the gray cell represents a byte that toggles at most once. In the 256-block CTR mode encryption, the AES encryption inputs $x_{1,14},\;x_{1,15}$ are expressed as follows:

(1) $$x_{1,15}=n_{15}+ctr\;\mathrm{mod}\;256\;\;\left(ctr\in \left\{0,1,2,\ldots ,255\right\}\right)$$

(2) $$x_{1,14}=n_{14}\;\mathrm{or}\;n_{14}+1.$$

The CTR mode characteristics enable it to recover secret keys using only power traces [26]. In addition, as Meyer stated [27], attacking the CTR mode without input and output information enables the SCA scenario against the CTR DRBG.

### 2.2. Correlation Power Analysis

CPA is an SCA technique that recovers fixed secret values using power traces generated by the fixed secret and variable known values [12]. We first calculate the hypothetical power consumption sequence $H_{k}$ using a fixed secret value *k* and a variable known values. Then, we calculate the PCC between the hypothetical power consumption sequence $H_{k}$ and the actual power consumption sequence **A**. The formula for the PCC $r_{A,H_{k}}$ between **A** and $H_{k}$ is given as follows, where cov is the covariance and $\sigma_{X}$ is the standard deviation of **X**:

(3) $$-1\leq r_{A,H_{k}}=\frac{\mathrm{cov}\left(A,H_{k}\right)}{\sigma_{A}\sigma_{H_{k}}}\leq 1.$$

In CPA, we use the absolute value of PCC $|r_{A,H_{k}}|$ for each guessed fixed secret *k*. We expect the highest absolute value of the PCC $|r_{A,H_{rk}}|$ for the correct guessing value rk.

We use Ratio as a performance indicator for CPA. Here, Ratio represents the value obtained by dividing the PCC of the correct guess by the highest PCC obtained with incorrect guesses. A Ratio value greater than 1.0 indicates a successful CPA, and a higher Ratio value indicates better CPA analysis performance. The computational complexity of the CPA is determined by multiplying the number of traces, the number of points in the power trace, and the number of cases of fixed secret values.

### 2.3. Clustering Algorithm

**Figure 4:**
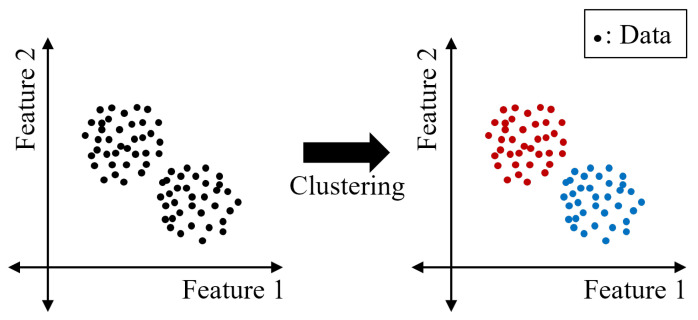
Clustering example.

Clustering, which is an unsupervised learning algorithm, groups a dataset with similar features. Figure 4 shows a simple example, where data with similar features are clustered into red and blue groups. In the power analysis, the data correspond to a power trace, and each feature represents the power consumption at different points. The clustering results of the power traces can be used to recover the secret information. Previous studies have explored SCA using clustering techniques [17,18,19].

The widely used K-means clustering algorithm iteratively updates the means and sets the number of clusters and iterations. In this study, we employ the K-means clustering algorithm for clustering. Algorithm 1 shows the pseudocode of the K-means clustering algorithm. The computational complexity of K-means clustering is determined by multiplying the number of clusters *U*, iterations *I*, data *T*, and the data dimensions of the $\vec{d_{t}}$. In power analysis using K-means clustering, the number of data equal the number of the power traces, and the data dimensions equal the number of points in the power trace.
**Algorithm 1** k-means clustering algorithm**Input:** data {$\vec{d_{1}},\vec{d_{2}},\ldots ,\vec{d_{T}}$}, number of clusters *U*, iteration *I***Output:** centroid {$\vec{c_{1}},\vec{c_{2}},\ldots ,\vec{c_{U}}$}, cluster {$S_{1},S_{2},\ldots ,S_{U}$}
   1:Initialize centroid {$\vec{c_{1}},\vec{c_{2}},\ldots ,\vec{c_{U}}$}   2:**for** iteration i=1 to *I* **do**   3:   $S_{1}=S_{2}=\cdots =S_{U}=\left\{\right\}$   4:   **for** data t=1 to *T* **do**   5:     **for** cluster u=1 to *U* **do**   6:        $S_{u}$ = ($\vec{c_{u}}$=closest centroid of $\vec{d_{t}}$)? $S_{u}\cup \left\{\vec{d_{t}}\right\}$:$S_{u}$   7:     **end for**   8:   **end for**   9:   **for** cluster u=1 to *U* **do** 10:     $\vec{c_{u}}=$ Mean($S_{u}$) 11:   **end for** 12:**end for** 13:**Return** {$\vec{c_{1}},\vec{c_{2}},\ldots ,\vec{c_{U}}$}, {$S_{1},S_{2},\ldots ,S_{U}$}


## 3. Previous Power Analysis on AES CTR Mode

In the following, we briefly explain Meyer’s power analysis on the AES CTR mode [27]. Meyer’s attack comprises four steps and uses 256 power traces generated during the encryption of each block in the AES CTR mode. In step 1, Meyer recovered 1 byte of the first round S-Box output using a 15-bit guessing CPA. In step 2, she recovered 4 bytes of the second round S-Box outputs using an 8-bit guessing CPA based on the byte recovered in step 1. Similarly, in step 3, she recovered all third-round S-Box outputs using the 8-bit guessing CPA. Finally, in step 4, she recovered all fourth-round keys. Subsequently, she recovered the master key of the AES using the fourth-round keys.

### 3.1. Attack Step 1

**Figure 5:**
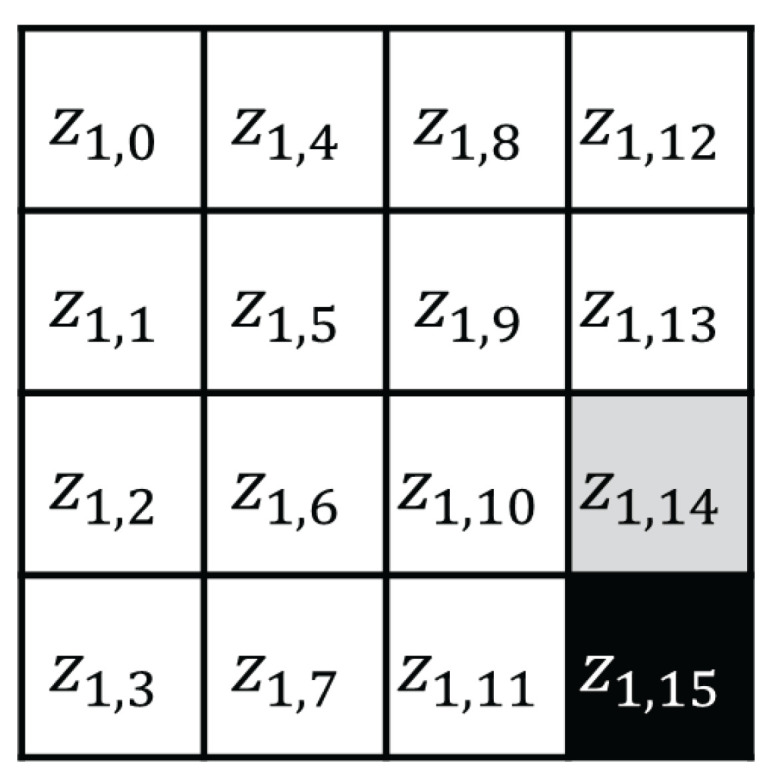
AES first SubBytes output state.

In step 1, we focus on the black cell in Figure 5. Here, $z_{1,15}$ is expressed as follows:

(4) $$z_{1,15}=S\left(k_{1,15}⊕x_{1,15}\right)=S\left(k_{1,15}⊕\left(n_{15}+ctr\;\mathrm{mod}\;256\right)\right)$$

Let $n_{15}=n_{15,hi}\left|\right|n_{15,lo}$ and $k_{1,15}=k_{1,15,hi}\left|\right|k_{1,15,lo}$, where hi denotes the most significant bit, and lo denotes the other 7 bits. In addition, let $b=n_{15,hi}⊕k_{1,15,hi}$. Then, $z_{1,15}$ can be expressed as follows:

(5) $$z_{1,15}=S\left(\left(b\ll 7\right)⊕k_{1,15,lo}⊕\left(n_{15,lo}+ctr\;\mathrm{mod}\;256\right)\right)$$

Thus, $z_{1,15}$ for each of the 256 traces can be recovered using 15-bit$\left(b,\;k_{1,15,lo},\;n_{15,lo}\right)$ guessing CPA.

### 3.2. Attack Step 2

**Figure 6:**
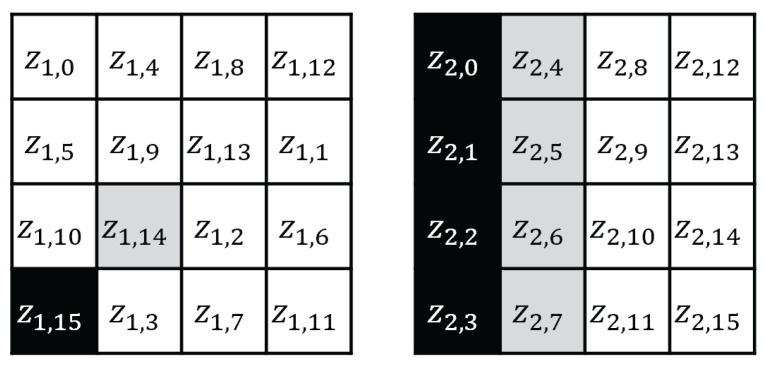
AES first ShiftRows and second SubBytes output state.

In step 2 of the attack, we recover $z_{2,0},\;z_{2,1},\;z_{2,2},\;z_{2,3}$, which are the black cells in the right of Figure 6. Here, $z_{2,0}$ is expressed as follows.

(6) $$z_{2,0}=S\left(\left(k_{2,0}⊕2\cdot z_{1,0}⊕3\cdot z_{1,5}⊕z_{1,10}\right)⊕z_{1,15}\right)$$

Note that $z_{1,15}$ is a known variable value (recovered in step 1), and $\left(k_{2,0}⊕2\cdot z_{1,0}⊕3\cdot z_{1,5}⊕z_{1,10}\right)$ is an unknown constant value. Thus, $z_{2,0}$ can be recovered using 8-bit$\left(k_{2,0}⊕2\cdot z_{1,0}⊕3\cdot z_{1,5}⊕z_{1,10}\right)$ guessing CPA, and $z_{2,1},z_{2,2},z_{2,3}$ can be recovered in a similar manner.

### 3.3. Attack Step 3

In attack step 3, we recover $Z_{3}$. $z_{3,0}$ as follows:

(7) $$z_{3,0}=S\left(\left(k_{3,0}⊕3\cdot z_{2,5}⊕z_{2,10}⊕z_{2,15}\right)⊕2\cdot z_{2,0}\right)$$

**Figure 7:**
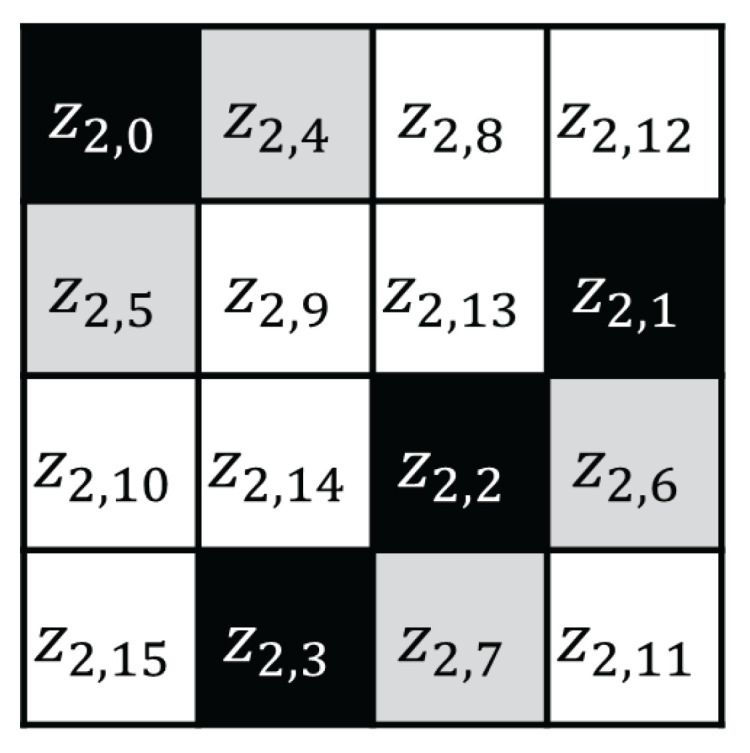
AES second ShiftRows output state.

Here, $z_{2,0}$ is a known value (recovered in step 2), and $\left(k_{3,0}⊕3\cdot z_{2,5}⊕z_{2,10}⊕z_{2,15}\right)$ is an unknown and nonconstant value (toggled by the $z_{2,5}$ value). In this case, $z_{3,0}$ is affected by the black and gray cells ($z_{2,0},z_{2,5}$, respectively) (Figure 7) and $z_{2,5}$ is toggled when counter carry occurs in the addition of $x_{1,15}=n_{15}+ctr$ mod 256. Meyer only recovered 7 bits of $n_{15}$ in step 1; thus, the index of the block in which $x_{14}$ is toggled is unknown. This is an obstacle to calculating the guessing value of $z_{3,0}$. In 256 traces, the values of $\left(k_{3,0}⊕3\cdot z_{2,5}⊕z_{2,10}⊕z_{2,15}\right)$ are divided into two different fixed values, and then the value of $z_{3,0}$ is affected by the different fixed values. Therefore, the accuracy performance of the CPA, which analyzes 256 traces by guessing the fixed value, is reduced.

Meyer discussed three cases, i.e., the best, average, and worst cases, for step 3 CPA. The best case does not involve the toggle (i.e., $n_{15}=0$), resulting in no interference to the CPA. The worst case occurs when the toggle occurs in approximately 128 traces, i.e., when $n_{15}\approx 128$. In this case, approximately half of the traces contribute noise to the CPA, thereby causing a significant reduction in its success rate. Meyer also proposed a CPA method that uses either the front 128 or back 128 traces to avoid a significant reduction in the CPA performance. However, CPA using 128 traces results in a reduced success rate, particularly in the best or average cases. In other words, each CPA method proposed by Meyer results in reduced CPA performance in some instances. $z_{3,0},z_{3,1},\ldots ,z_{3,15}$ can be recovered using 8-bit guessing CPA using the 256-trace or 128-trace.

### 3.4. Attack Step 4

In step 4, we recover the $K_{4}$ and AES master keys. $X_{4}$ can be calculated using $Z_{3}$ recovered in step 3. $K_{4}$ can then be recovered using 8-bit guessing CPA 16 times. Here, similar to step 3, the CPA accuracy performance decreases. Finally, the master key of the AES can be calculated using $K_{4}$.

## 4. Our Proposed Power Analysis on AES CTR Mode

This section proposes the power analysis for the AES CTR mode. The proposed attack comprises four steps and uses 256 power traces, the same as Meyer’s attack. However, in contrast to Meyer’s approach, we introduce a clustering algorithm in step 1 to recover more information more accurately. In step 1, while Meyer recovered 7 bits of the input and key, we recover the 1-byte input by clustering, and then the 1-byte key is recovered through 8-bit guessing CPA. Note that step 2 remains the same as in Meyer’s attack. Finally, we improve the 8-bit guessing CPA in steps 3 and 4 using the additional information obtained in step 1.

### 4.1. Attack Step 1

The first step is crucial in both Meyer’s attack and the proposed attack. If the first step fails, all subsequent steps of the attack will also fail. In addition, the first step is the most complex in terms of the computation and the attack complexity.

Rather than using 15-bit guessing CPA, we perform a single clustering attack and a single 8-bit guessing CPA. The clustering process divides the 256 power traces into two clusters and recovers $n_{1,15}$ using the results. We then guess the 8-bit $k_{1,15}$ and perform CPA on $z_{1,15}$ to recover $k_{1,15}$ (Equation (4)). This result recovers $\left(n_{15},k_{1,15}\right)$ rather than $\left(b=n_{15,hi}⊕k_{1,15,hi},n_{15,lo},k_{1,15,lo}\right)$. Theoretically, the 15-bit guessing CPA has a lower attack success rate than the 8-bit guessing CPA; thus, the success rate of the clustering attack is very important in the proposed attack. We describe the clustering attack method and several ways to improve the attack success rate.

**Figure 8:**
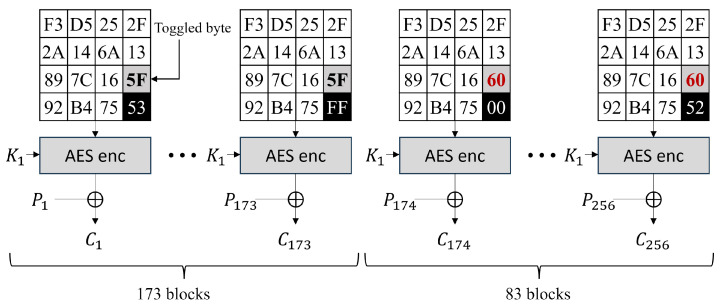
Example of 256-block encryption and *toggled byte* of CTR mode.

Here, we focus on the byte of the AES state being toggled (i.e., the gray cells in the AES state figures). We defined the *toggled byte* as the byte to be toggled when it causes carry in $x_{1,15}$, i.e., the gray cell in the AES state figures. The *toggled byte*s contain $x_{1,14},x_{2,4},x_{2,5},x_{2,6},x_{2,7},z_{2,4},z_{2,5},z_{2,6},z_{2,7}$, and others. Figure 8 shows an example of the 256-block encryption of the AES CTR mode when $n_{1}5=0x53$ and $n_{1}4=0x5f$. We use the difference in the power consumptions of the *toggled byte*. In Figure 8, the forward 173 blocks have different power consumptions with the backward 83 blocks in computing the *toggled byte*. If we can classify the power consumption into two groups, we can recover the $n_{1}5$ value, and the clustering algorithm is one of the solutions that classify the power consumption into two groups. Here, the clustering algorithm is used to determine the power consumption of the *toggled byte*s (i.e., the gray cells in the AES state figures). Note that the type of analysis result varies depending on the following two cases.

**Case 1. $n_{15}=\alpha \left(\alpha \neq 0\right)$**

The *toggled byte*s remain constant in the front 256−α and back α traces. Therefore, we predict that the power consumption while operating the *toggled byte* of the front 256−α traces and the back α traces will exhibit significantly different distributions. Here, if we can successfully cluster the front 256−α and back α traces, then backwards, we can recover N=α using the clustering results.

**Case 2. $n_{15}=0$**

Note that the *toggled byte*s are constant in all 256 traces; thus, the power consumption while operating the *toggled byte* of all 256 traces will exhibit the same distributions. Therefore, applying a clustering algorithm will not yield clear clustering results. However, backwards, we can know that N=0 through the unclear clustering results.

In addition, we can recover $n_{15}$ using the clustering results of the 256 traces. However, if the value of $n_{15}$ is close to 0 or 255, the clustering accuracy may be affected by the number of data in each set, and this limitation can be overcome using an optimized clustering algorithm or data augmentation technique, e.g., adding copies of the 0th and 255th power traces to the dataset. The data in this attack possess characteristics that can optimize the clustering process. First, the 256 traces are always clustered into two classes. Second, consecutive data points fall into the same cluster before and after the toggle point. Third, the *toggled byte*s exist within the AES traces at multiple points in time. Note that the first and second characteristics simplify the clustering problem and imply the existence of a more optimized clustering algorithm for this problem. The third characteristic allows securing more data features, which means that the clustering success rate can be improved through multiple feature information even in a higher noise environment. Thus, the attack can be improved by applying a clustering algorithm that uses these data characteristics effectively. In the experiments conducted in this study (Section 5), we employed power traces for part of the *toggled byte*s and non-modified K-means clustering, which outperformed both the 8-bit and 15-bit guessing CPA.

Next, with the knowledge of $x_{1,15}$ through $n_{15}$, we can recover $k_{1,15}$, $z_{1,15}=S\left(x_{1,15}⊕k_{1,15}\right)$ using 8-bit guessing CPA. The difference from Meyer’s attack is that we know the exact toggle point, i.e., $n_{1}5$, and this advantage is exploited in the CPA of attack steps 3 and 4.

### 4.2. Attack Step 2

In step 1, we recovered additional information about *toggled byte*s (gray cells). However, the target values $z_{2,0},\;z_{2,1},\;z_{2,2},\;z_{2,3}$ of step 2 are not related to the *toggled byte*s. Therefore, we follow the same attack described in Section 3.2.

### 4.3. Attack Step 3

$z_{3,0}$ is expressed in Equation (7). Here, we have recovered all 8 bits of $n_{15}$ in step 1, so we know exactly when $z_{2,5}$ is toggled, unlike Section 3.3. Thus, we can divide the 256 traces into two sets based on the $z_{2,5}$ value. We can prevent the degradation of CPA accuracy performance due to traces acting as noise mentioned in Section 3.3 by performing CPA on traces classified with the same value of $z_{2,5}$. Although we still do not know the value of $z_{2,5}$, we optimize the CPA by using the maximum number of traces for which $z_{2,5}$ is fixed, thereby minimizing the accuracy degradation.

Meyer’s approach performs well with 256-trace CPA in the best case ($n_{15}=0$) and with 128-trace CPA in the worst case ($n_{15}=128$). On the other hand, our optimized CPA method always uses the maximum number of traces about a fixed $z_{2,5}$ value, so it is optimized for all cases. For example, when $n_{15}=192$, Meyer’s 256-trace CPA has 64 traces acting as noise. However, in the proposed attack, we can perform optimized CPA without any traces acting as noise in CPA by using only 192 traces with fixed $z_{2,5}$ as the same value. Similarly, we can recover $z_{3,0},\;z_{3,1},\ldots ,z_{3,15}$ via the optimized 8-bit guessing CPA.

### 4.4. Attack Step 4

In step 4, we perform 8-bit guessing CPA on $z_{4,j}=S\left(k_{4,j}⊕x_{4,j}\right)$ to recover $K_{4}$ and the AES master key. As discussed in Section 3.4, the CPA target $Z_{4}$ is calculated from $Z_{3}$; thus, the same CPA accuracy degradation as in step 3 occurs. As described in Section 4.3, we can perform the optimized CPA using the maximum number of traces in which $Z_{3}$ is fixed.

## 5. Experimental Results for the Proposed Clustering Attack

This section describes the experimental results for the clustering attack of the proposed attack first step.

**Figure 9:**
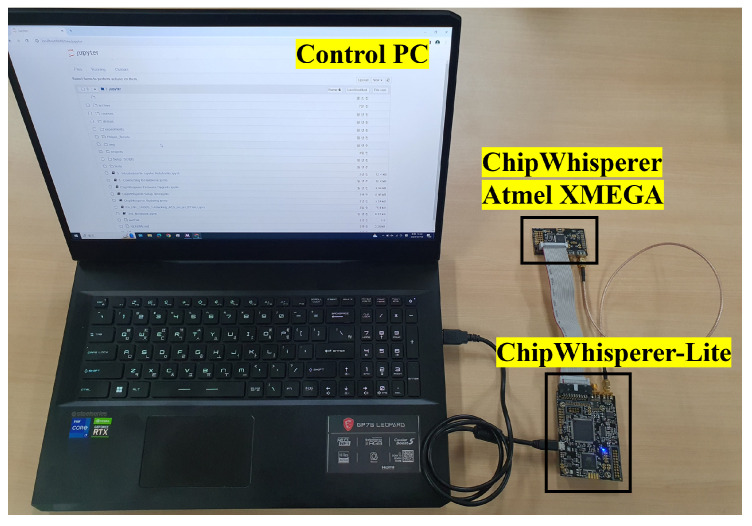
Experimental setup.

Figure 9 shows the experimental setup used to measure the power traces from the Atmel XMEGA target using ChipWhisperer-Lite. Table 1 shows the details of the experimental setup. We selected this setting with a low noise level and added artificial noise to evaluate the attack success rate at various noise levels. This section discusses the attacks performed at the default noise level, and Section 6 discusses the attacks performed at various noise levels. We experimentally verified the effectiveness of the proposed clustering attack described in Section 4.1. The attack was performed by collecting power consumption while encrypting 256 blocks using the AES-128 CTR mode on the Atmel XMEGA128D4 processor.

**Figure 10:**
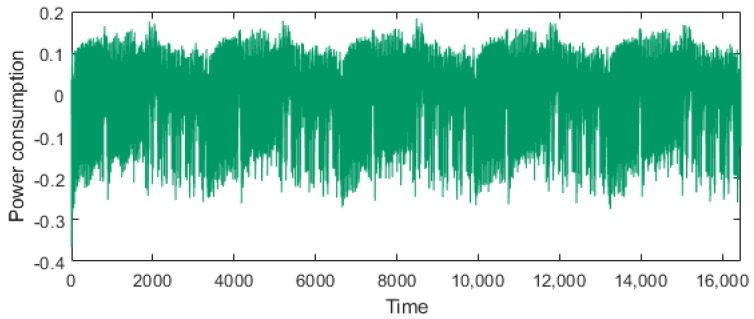
Power trace of AES for five rounds.

Figure 10 shows the power trace during AES execution from the first to the fifth round. As discussed in Section 4.1, the power consumption suitable for clustering is the part where the *toggled byte* is manipulated. The first and second rounds involve the *toggled byte*s in the AddRoundKey, SubBytes, ShiftRows, and Mixcolumns operations. In these experiments, we used a power consumption range of 4400–4500 points, which is part of the second round of the SubBytes operation. Here, we employed the K-means clustering algorithm with two clusters and one iteration.

**Figure 11:**
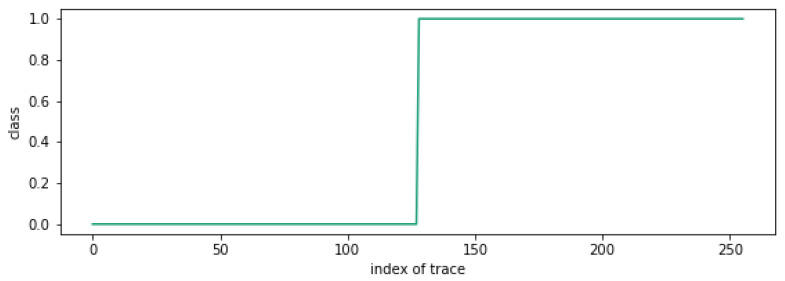
Clustering attack result when $n_{15}=128$.

Figure 11 shows the clustering results for 256 traces with $n_{15}=128$, where the *x*-axis represents the trace index, and the *y*-axis represents the corresponding cluster number assigned to each trace. The graph shows a toggled point at the 128th trace. Thus, we can recover $n_{15}$ as $n_{15}=256-128=128$.

**Figure 12:**
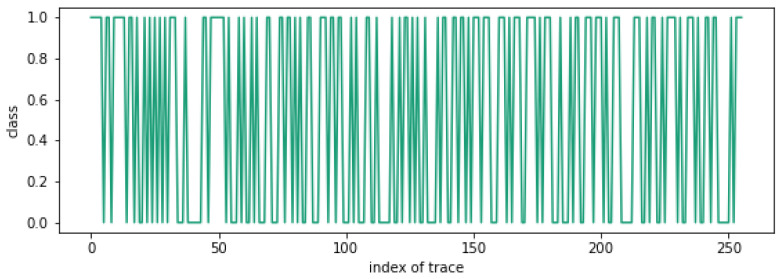
Clustering attack result when $n_{15}=0$.

Figure 12 shows the clustering results for 256 traces with $n_{15}=0$. As can be seen, the clustering result is unclear. Therefore, we can recover $n_{15}$ as $n_{15}=0$.

## 6. Comparison of Previous and Proposed Attacks

In this section, we compare the proposed attack with Meyer’s attack. Regarding step 1 of the attack, we compare the attack success rate based on the noise level and computational complexity. In addition, we compare the CPA accuracy performance for steps 3 and 4 of the attack.

### 6.1. Comparison of Attack Step 1

#### 6.1.1. Attack Success Rate

**Figure 13:**
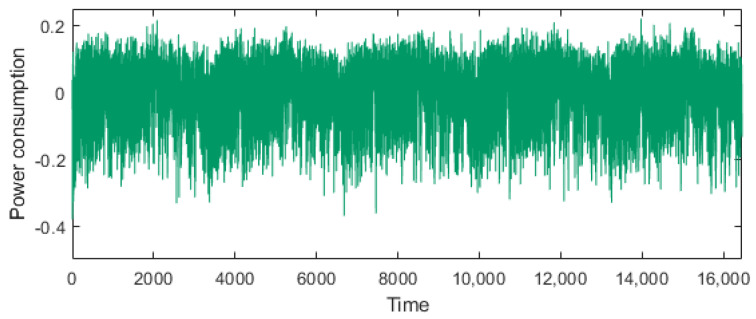
Power trace of AES for five rounds with inserted Gaussian noise.

Gaussian noise with an average of 0 was added to the experimental data described in Section 5. These data, were then used as the experimental data to compare the attack success rate based on the noise level. Here, Gaussian noise with a random multiple of the sample standard deviation was added for each point. Figure 13 shows a power trace with the added Gaussian noise, where the noise level is set to nine times the standard deviation of the sample for each time point derived from the power trace discussed in Section 5.

We selected $n_{15}=128$ and defined that the clustering attack was successful when the class was first changed at the index of 128. We also defined the CPA as successful when the correct guess had the highest correlation coefficient. We determined that step 1 of the proposed attack was successful when the clustering attack and 8-bit CPA were both successful.

To compare the success rate of step 1 of the proposed attack, we generated 256 power traces for each noise level, repeated this process ten times, and conducted an attack on each dataset. For the clustering attack, we used the power trace of the range of 4400–4500 points, which corresponds to the SubBytes operation in the second round. For CPA, we used a data point range of 1000–3200, encompassing the SubBytes to Mixcolumns operations in the first round.

**Figure 14:**
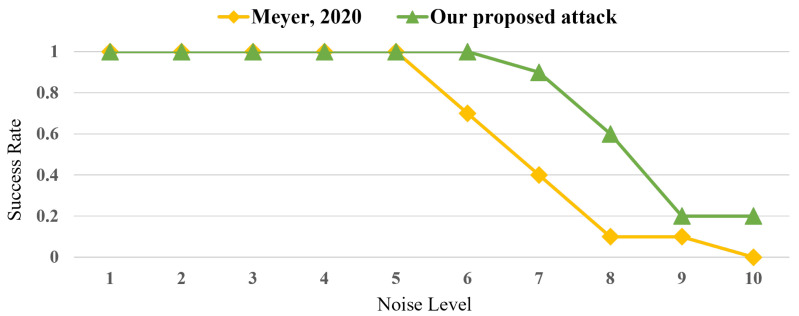
Success rate result of step 1 of Meyer’s attack [27] and the proposed attack according to the noise level.

Figure 14 compares the success rate of step 1 of each attack according to the noise level. The noise level indicates the number of times the standard deviation of the inserted Gaussian noise was equal to that of the sample of the existing power trace. As can be seen, Meyer’s attack exhibits a significant decline at noise level 6, and its success rate is 0% at noise level 10. However, the success rate of the proposed attack decreases at noise level 7, and its success rate is 20% at noise level 10. In particular, the success rate of the proposed attack in step 1 is higher than that of Meyer’s attack by a maximum of 50% at high noise levels (i.e., noise levels 7 and 8).

#### 6.1.2. Attack Complexity

Step 1 of Meyer’s attack performs a 15-bit guessing CPA and has the highest computational complexity among all steps. In contrast, step 1 of the proposed attack performs clustering and 8-bit guessing CPA. The computational complexity of the CPA and K-means clustering algorithm are described in Section 2.2 and Section 2.3, respectively. The number of cases of the fixed secret values is $2^{n}$ in the *n*-bit guessing CPA, and the number of clusters *U* is fixed to 2 in this attack. In addition, if the number of traces is *T*, the number of points is *P*, and the number of clustering iterations is *I*, the computational complexity of step 1 of each attack can be determined as follows:

$$\mathrm{Meyer}’s\;\mathrm{attack}\;\mathrm{step}\;1\;:\;O(T\times P\times 2^{15})$$

$$\mathrm{Proposed}\;\mathrm{attack}\;\mathrm{step}\;1\;:\;O\left(T\times P\times 2\times I\right)+O\left(T\times P\times 2^{8}\right)$$

If the number of iterations is set to 1, as in the experiments described in Section 5 and Section 6.1.1, the computational complexity of step 1 in the proposed attack is approximately 1/128 that of Meyer’s attack.

### 6.2. Comparison on Steps 3 and 4 of the Attack

Meyer proposed two types of CPA using 128 or all 256 traces [27]; however, in contrast, the proposed attack enables the recovery of all bits of $n_{15}$ in step 1, which allows us to perform CPA using the maximum number of traces where the unknown value is fixed.

**Figure 15:**
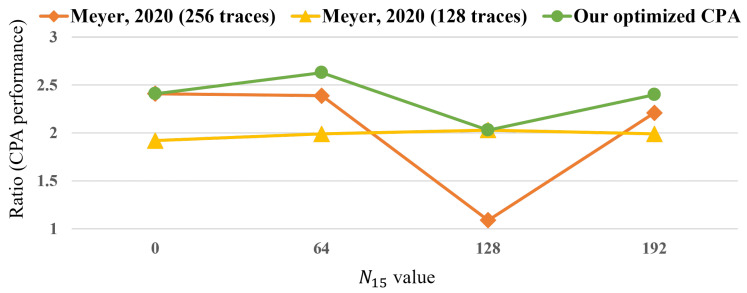
Performance of Meyer’s CPA [27] and the optimized CPA in steps 3 and 4 of the attack.

Figure 15 compares the Ratio, which represents the CPA performance, between Meyer’s and the proposed CPA in steps 3 and 4, using the experimental environment described in Table 1. Here, we performed CPA and computed the Ratio for $n_{15}$ values of 0, 64, 128, and 192. Note that our optimized CPA employs the maximum number of traces where the unknown value is fixed; thus, our CPA exhibited the highest Ratio in all cases. On average, the Ratio of the CPA using 256 traces was 2.03, that of CPA using 128 traces was 1.98, and the optimized CPA had a Ratio value of 2.37. As a result, the optimized CPA demonstrated an average performance improvement of 18.5% compared to the method proposed by Meyer.

## 7. Conclusions

This paper has proposed an improved power analysis method for the AES CTR mode based on Meyer’s previous approach. In addition, we have demonstrated the effectiveness of the proposed attack method based on actual experiments. In a high-noise experimental environment, we found that the proposed attack outperforms Meyer’s attack, achieving up to a 50% higher success rate in the first stage, and the computational complexity was reduced. The CPA performance improved by approximately 18.5% in steps 3 and 4 of the attack. In addition, the clustering process in the proposed attack can be optimized further using the characteristics of the AES CTR mode. These results demonstrate that the proposed attack extended the target device to more noisy environments than previous attacks, thereby increasing the threat of SCAs on the CTR DRBG.

As described in the literature [27], these attacks (the proposed attack and Meyer’s attack) were designed to target the CTR mode, which uses addition as a counter and thus can not be applied to the CTR mode using a linear-feedback shift register (LFSR) as a counter. In addition, the limitation of the request size of CTR DRBG can provide resistance for these attacks by reducing the number of traces for the attack. However, these attacks arise from the relationship between each counter block; thus, the CTR mode with an LFSR counter and the limitation of the request size could still be susceptible to as-yet undiscovered attacks. Conservatively, we expect that it would be more effective from a security perspective to employ a counter that makes it difficult to guess the relevance of each counter block, e.g., a hash function.

In addition, attacks targeting hardware implementations, such as those on FPGAs or ASICs, remain an open area for future research. Both Meyer’s and our proposed attacks operate with only 256 power traces in a non-profiled setting, requiring the success of all four steps to fully recover secret information. In practice, however, hardware implementations (e.g., FPGAs, ASICs) typically exhibit higher noise levels than MCUs, making such attacks more difficult to succeed. Therefore, to perform practical attacks on hardware implementations of CTR DRBG, future research should explore conditions that allow for profiling or access to a larger number of power traces, as demonstrated in Tienteu’s work. Additionally, since hardware implementations tend to conform more closely to the Hamming distance leakage model, adapting the attack logic to accommodate this characteristic is also expected to be necessary. 

## Figures and Tables

**Table 1:** Experimental environment.

Power Measurement Board	ChipWhisperer-Lite
Target chip	Atmel XMEGA128D4
Number of AES traces	256
Sampling rate	29.538 MS/s

## Data Availability

The original traces and noisy traces used in our demonstrations are available from the following link: https://drive.google.com/file/d/1O6vo7QFC13HIWwCKl0ss0VE6ksCNgzwN/view (accessed on 28 May 2025).

## Abbreviations

The following abbreviations are used in this manuscript:

SCA	Side-Channel Attack
CPA	Correlation Power Analysis
CTR	Counter
DRBG	Deterministic Random Bit Generator

## Notations

The notations used in this paper are defined as follows:

⊕	Exclusive OR operator
≪	Shift operator in 8-bit
·	AES field multiplication operator
S()	AES S-Box function
*i*	Round index (i∈{1,2,…,10})
*j*	Byte index (j∈{0,1,…,15})
$X_{i}$	*i*-th round input 16 bytes
$x_{i,j}$	*i*-th round input *j*-th byte
$K_{i}$	*i*-th round key 16 bytes
$k_{i,j}$	*i*-th round key *j*-th byte
$Z_{i}$	*i*-th round SubBytes output 16 bytes
$z_{i,j}$	*i*-th round SubBytes output *j*-th byte
*N*	Nonce value 16 bytes of CTR mode
$n_{j}$	Nonce value *j*-th byte of CTR mode
ctr	Counter value of CTR mode
